# An Auristatin nanoconjugate targeting CXCR4+ leukemic cells blocks acute myeloid leukemia dissemination

**DOI:** 10.1186/s13045-020-00863-9

**Published:** 2020-04-15

**Authors:** Victor Pallarès, Ugutz Unzueta, Aïda Falgàs, Laura Sánchez-García, Naroa Serna, Alberto Gallardo, Gordon A. Morris, Lorena Alba-Castellón, Patricia Álamo, Jorge Sierra, Antonio Villaverde, Esther Vázquez, Isolda Casanova, Ramon Mangues

**Affiliations:** 1 Biomedical Research Institute Sant Pau (IIB-Sant Pau), Hospital de la Santa Creu i Sant Pau, 08041 Barcelona, Spain; 2 Josep Carreras Research Institute, 08041 Barcelona, Spain; 3 CIBER en Bioingeniería, Biomateriales y Nanomedicina (CIBER-BBN), 08041 Barcelona, Spain; 4 Departament de Genètica i de Microbiologia, Universitat Autònoma de Barcelona, 08193 Bellaterra, Spain; 5 Institut de Biotecnologia i de Biomedicina, Universitat Autònoma de Barcelona, 08193 Bellaterra, Spain; 6 Department of Pathology, Hospital de la Santa Creu i Sant Pau, Barcelona, Spain; 7 Department of Chemical Sciences, School of Applied Sciences, University of Huddersfield, Huddersfield, HD1 3DH UK; 8 Department of Hematology, Hospital de la Santa Creu i Sant Pau, Barcelona, Spain

**Keywords:** Acute myeloid leukemia, CXCR4, Targeted nanoparticle, Auristatin nanoconjugate, Leukemic stem cells, Disseminated AML mouse model

## Abstract

**Background:**

Current acute myeloid leukemia (AML) therapy fails to eliminate quiescent leukemic blasts in the bone marrow, leading to about 50% of patient relapse by increasing AML burden in the bone marrow, blood, and extramedullar sites. We developed a protein-based nanoparticle conjugated to the potent antimitotic agent Auristatin E that selectively targets AML blasts because of their CXCR4 receptor overexpression (CXCR4+) as compared to normal cells. The therapeutic rationale is based on the involvement of CXCR4 overexpression in leukemic blast homing and quiescence in the bone marrow, and the association of these leukemic stem cells with minimal residual disease, dissemination, chemotherapy resistance, and lower patient survival.

**Methods:**

Monomethyl Auristatin E (MMAE) was conjugated with the CXCR4 targeted protein nanoparticle T22-GFP-H6 produced in *E. coli*. Nanoconjugate internalization and *in vitro* cell viability assays were performed in CXCR4+ AML cell lines to analyze the specific antineoplastic activity through the CXCR4 receptor. In addition, a disseminated AML animal model was used to evaluate the anticancer effect of T22-GFP-H6-Auristatin in immunosuppressed NSG mice (*n* = 10/group). *U* of Mann-Whitney test was used to consider if differences were significant between groups.

**Results:**

T22-GFP-H6-Auristatin was capable to internalize and exert antineoplastic effects through the CXCR4 receptor in THP-1 and SKM-1 CXCR4+ AML cell lines. In addition, repeated administration of the T22-GFP-H6-Auristatin nanoconjugate (9 doses daily) achieves a potent antineoplastic activity by internalizing specifically in the leukemic cells (luminescent THP-1) to selectively eliminate them. This leads to reduced involvement of leukemic cells in the bone marrow, peripheral blood, liver, and spleen, while avoiding toxicity in normal tissues in a luminescent disseminated AML mouse model.

**Conclusions:**

A novel nanoconjugate for targeted drug delivery of Auristatin reduces significantly the acute myeloid leukemic cell burden in the bone marrow and blood and blocks its dissemination to extramedullar organs in a CXCR4+ AML model. This selective drug delivery approach validates CXCR4+ AML cells as a target for clinical therapy, not only promising to improve the control of leukemic dissemination but also dramatically reducing the severe toxicity of classical AML therapy.

## Background

C-X-C chemokine receptor type 4 (CXCR4) receptor overexpression (CXCR4+) associates with dissemination, relapse, minimal residual disease, and poor survival in acute myeloid leukemia (AML) patients [1–4]. Moreover, CXCR4+ leukemic blasts become anchored and quiescent in the bone marrow (BM) through CXCR4-CXCL12 (C-X-C motif chemokine 12 or stromal cell-derived factor 1; SDF1) interaction [5], displacing normal hematopoietic stem cells (HSCs) to cause patient death [6]. CXCR4+ AML cells also circulate in the blood [4] to reach extramedullar CXCL12 producing sites [4, 7], mainly the liver and spleen [5, 7–11]. All these functions support CXCR4+ AML cells as Leukemic stem cells (LSCs), despite the lack of a formal proof. Similarly, CXCR4+ cancer cells are metastatic stem cells in several solid tumors [12–17]. Consequently, different groups are developing small drug CXCR4 inhibitors for AML therapy [5, 7, 18] aimed at eliminating CXCR4+ LSCs, responsible for AML aggressiveness and patient death.

Nanotechnological approaches can increase selectivity regarding target cancer cell killing, while reducing systemic toxicity [19]. Here, we specifically tested if a novel T22-GFP-H6-Auristatin nanoconjugate (NC) selectively eliminates CXCR4+ AML cells, and therefore LSCs, in an AML disseminated model, to improve chemotherapy performance, which reduces the differentiated tumor bulk but does not eliminate LSCs [20]. The NC incorporates a protein nanoparticle to selectively internalize in CXCR4+ AML cells by exploiting high CXCR4 overexpression in LSCs as compared to HSCs [6, 20], similarly to a previous approach in colorectal cancer (CRC) [21, 22]. The NC is loaded with Auristatin E, a microtubule-destabilizing toxin, introduced in clinical onco-hematology [23, 24]. This drug eliminates quiescent cancer cells [23, 24], overcoming the limited effectiveness of current AML chemotherapy, unable to kill LSCs in the G0 phase [6, 20].

We expected the novel T22-GFP-H6-Auristatin NC to selectively deliver Auristatin E to cycling and quiescent CXCR4+ LSCs target cells achieving a potent inhibition of AML dissemination in the absence of toxicity, because of its low uptake in normal tissues, as we reported for a NC loaded with a CRC sensitive drug [22]. To that aim, we generated a bioluminescent CXCR4+ disseminated AML model, which allows a non-invasive follow-up of the response in BM, liver, and spleen, the clinically relevant sites. We describe CXCR4-dependent internalization and killing of CXCR4+ AML cells for this novel NC in vitro. We also report in this in vivo model, a potent antineoplastic effect on BM and blockade of dissemination to extramedullar sites.

## Methods

### Drugs, nanoparticle, and nanoconjugate

Monomethyl Auristatin E (MMAE) is a potent tubulin polymerization inhibitor that was acquired by custom systhesis from Levena Biopharma (Levena Biopharma, San Diego, CA, USA) as a maleimide conjugated MMAE (MC-MMAE). T22-GFP-H6 is a CXCR4 targeted protein nanoparticle produced in *E. coli* as previously described [21]. T22-GFP-H6-Auristatin nanoconjugates were synthesized by covalent binding of the targeting vector (T22-GFP-H6) with the therapeutic moiety (MC-MMAE). For that, an excess of MC-MMAE was incubated with T22-GFP-H6 nanoparticles and reacted with amino groups of external lysines in a 1:50 ratio (protein to MC-MMAE) for 4 h at room temperature. T22-GFP-H6-Auristatin nanoconjugates were then again purified by IMAC affinity chromatography using HiTrap Chelating HP 5 mL columns in an ÄKTA pure (GE Healthcare, Chicago, IL, USA) in order to remove non-reacted free MC-MMAE. Finally, re-purified nanoconjugates were dialyzed against sodium carbonate buffer (166 mM NaCO_3_H, 333 mM NaCl pH = 8) and conjugation efficiency and presence of free MMAE checked by MALDI-TOF mass spectrometry.

The volume size distribution of T22-GFP-H6 nanoparticles and resulting nanoconjugates (T22-GFP-H6-Auristatin) was determined by dynamic light scattering at 633 nm in a Zetasizer Nano (Malvern Instruments, Malvern, UK). Measurements were performed in triplicate. In addition, ultrastructural morphometry of T22-GFP-H6-Auristatin nanoconjugates (size and shape) was determined at nearly native state with field emission scanning electron microscopy (FESEM). Samples were directly deposited on silicon wafers (Ted Pella Inc., Redding, CA, USA) for 30 s, excess of liquid blotted, air dried, and immediately observed without coating with a FESEM Zeiss Merlin (Zeiss, Oberkochen, Germany) operating at 1 kV and equipped with a high resolution in-lens secondary electron detector. Representative images of a general field were captured at two high magnifications (× 100,000 and × 120,000). In a quantitative approach, nanoconjugates average size from FESEM images were analyzed by Image J software (1.8.0.172, National Institutes of Health, USA) [25].

The average molar mass of T22-GFP-H6 nanoparticles and T22-GFP-H6-Auristatin nanoconjugates was measured by a size exclusion chromatography coupled to a multi-angle light scattering (SEC-MALS). Samples were injected in a Superdex 200 increase 10/300 GL column (GE Healthcare, Chicago, IL, USA) and run in a degassed sodium carbonate buffer with Nickel (166 mM NaCO_3_H, 333 mM NaCl, 0.1 mM NiCl_2_ pH = 8). The eluent was monitored by an in-line UV-Vis detector, a Dawn Heleos MALS detector and an Optilab rEX RI detector (Wyatt Technology Corporation, Santa Barbara, CA, USA). All data were analyzed using Astra 6.0.2.9 software (Wyatt Technology Corporation). Molecular weights were double-checked from MALS with UV and RI signals using ASTRA software and dn/dc (mL/g) values of 0.185 and UV extinction Coefficient (mL/(mg.cm)) values of 1.099 for all proteins. Conjugated MMAE ratio was calculated by subtracting the average molecular weight of T22-GFP-H6 nanoparticles to the average molecular weight of T22-GFP-H6-Auristatin nanoconjugates and dividing by the molecular weight of MMAE molecule.

Fluorescence of T22-GFP-H6 nanoparticles and T22-GFP-H6-Auristatin nanoconjugates was determined in a Cary Eclipse fluorescence spectrophotometer (Varian Inc, Palo Alto, CA, USA) at 510 nm upon excitation at 488 nm.

T22-GFP-H6-Auristatin nanoconjugates proteolytic stability in serum was determined by western blot immunostaining using a monoclonal anti-His antibody (Santa Cruz Biotechnology, Dallas, TX, USA) upon incubation in front of human serum (Sigma-Aldrich, St-Louis, MO, USA) at different time points (0, 2, 5, and 24 h) at 37 °C and a final concentration of 1 mg/mL.

### Cell lines

The AML cell lines used in this study were purchased from DSMZ (Leibniz Institute DSMZ-German Collection of Microorganisms and Cell Cultures, Braunschweig, Germany). THP-1 and SKM-1 were cultured in RPMI-1640 medium supplemented with 10% FBS, 10 mmol/L L-glutamine, 100 U/mL penicillin, 10 mg/mL streptomycin and 0.45 μg/mL fungizone (Gibco, Thermo Fisher Scientific (TFS), Waltham, MA, USA). Cells were kept at 37 °C in a humidified atmosphere of 5% CO_2_. THP-1 cells were transfected with a plasmid encoding the luciferase gene that confers bioluminescence (BLI) to the cells using Lipofectamine LTX and PLUS reagents (A12621, Invitrogen, TFS) according to the manufacturer’s instructions.

### Quantitation of CXCR4 membrane levels in AML cell lines

To detect cell surface expression of CXCR4 in AML cell lines, fluorescence-activated cell sorting (FACS) analysis was performed as described before [26]. Two technical and three biological replicates were performed. Data acquisition was analyzed by Cell Quest Pro software (BD Biosciences, San Jose, CA, USA) and results were expressed as mean fluorescence intensity (MFI) ± standard error (SE).

### Antineoplastic effect and internalization analyses of T22-GFP-H6-Auristatin in CXCR4+ AML cells in vitro

To assess the antineoplastic effect of the nanoparticle T22-GFP-H6 and the nanoconjugate T22-GFP-H6-Auristatin in AML cell lines, XTT cell viability assays were performed. Cells were cultured at 20 × 10^4^ cells/mL for 24 h in 96-well plates. After that, T22-GFP-H6-Auristatin was added in a range of 30–300 nM and T22-GFP-H6 at 500 nM. Forty-eight hours later, cells were incubated for 4 h with the XTT reagents (Cell Proliferation Kit II, Roche Diagnostics, Basel, Switzerland) and then the absorbance was measured at 492 nm in a FLUOstar OPTIMA spectrophotometer (BMG Labtech, Ortenberg, Germany). Five technical and three biological replicates were performed for each condition. The growth inhibitory activity was obtained by subtracting the absorbance of the blanks and expressed as the percentage of cell growth inhibition (± SE), as compared with untreated controls.

To find out the mechanism of cell death mediating T22-GFP-H6-Auristatin antineoplastic activity in AML cells, nuclear staining with DAPI dye in THP-1 and SKM-1 cells exposed to 240 nM T22-GFP-H6-Auristatin or buffer for 24 or 48 h, in 12-well plates (50 × 10^4^ cells/mL), was performed. After incubation, the media was collected and centrifuged to obtain the suspended cells. Cells were rinsed once with PBS. Afterwards, they were centrifuged and fixed (3.7% p-formaldehyde in PBS, pH= 7.4) for 10 min at − 20 °C, washed with PBS, and resuspended in 10 μL of PBS. Finally, cells were mounted on a slide with DAPI dye (ProLong™ Gold Antifade Mountant with DAPI, P36935, Invitrogen, Carlsbad, CA, USA) and observed for the appearance of the apoptotic bodies or mitotic catastrophe (MC), as induced events, under a fluorescence microscope (Olympus BX53, Olympus, Tokyo, Japan). Cell death events such as MC or apoptosis were quantified using the point tool in Image J software (1.8.0.172) and 4 randomly chosen fields for each condition.

To evaluate the internalization capacity of the nanoconjugate T22-GFP-H6-Auristatin in AML cell lines, FACS analyses were performed as described before [26] exposing cells to the vehicle or with T22-GFP-H6-Auristatin between 30 and 300 nM. Two technical and three biological replicates were performed for each condition. Data acquisition was analyzed by Cell Quest Pro software (BD Biosciences) and results were expressed as MFI ± SE.

Receptor competition assays were performed with the antagonist of CXCR4, AMD3100. To that aim, internalization analyses or XTT cell viability assays were performed as described above, treating cells with 240 nM of T22-GFP-H6-Auristatin with or without a pre-treatment for 1 h of AMD3100 (2000 nM).

### Evaluation of the antineoplastic effect of T22-GFP-H6-Auristatin in a disseminated CXCR4+ AML mouse model

NSG (NOD-*scid* IL2Rgamma^null^) female mice (4 weeks old) were obtained from Charles River Laboratories (Wilmington, MA, USA). Mice were housed in microisolator units with sterile food and water ad libitum. All procedures were conducted in accordance with the guidelines approved by the institutional animal Ethics Committee of Hospital Sant Pau. Three healthy (not having cancer) and untreated mice (not administered with vehicle or nanoconjugate) were assigned to the Normal mouse group (normal, *n* = 3). To generate the AML mouse model, 19 mice were intravenously (IV) injected with luciferase-transfected THP-1 cells (THP-1-Luci; 1 × 10^6^ cells/200 μL). These mice were divided randomly into two different experimental groups (day 0) (Fig. 5). One group (VEH; *n* = 10) was IV injected (Day 2) with the vehicle of the nanoconjugate (Buffer NaCO_3_H + NaCl pH = 8) and another group (T22-AUR; *n* = 9) with 100 μg of T22-GFP-H6-Auristatin daily for a total of 9 doses. The evolution of AML dissemination in mice was monitored using the IVIS Spectrum Imaging System (PerkinElmer, Waltham, MA, USA) three times per week until the day of the euthanasia. Animal weight was measured the same day as that of BLI analysis. All mice were euthanized the day that the first animal presented relevant signs of disease such as lack of mobility or 10% weight loss. Animals were intraperitoneally injected with luciferin; after 5 min, blood was obtained by intracardiac extraction, and mice were killed by cervical dislocation. Blood samples were collected for BLI analysis in IVIS and CD45-positive cells detection by flow cytometry. Tissues were excised to analyze the BLI levels ex vivo, and preserved in paraffin for further histological and immunohistochemical analyses. BLI measurements were expressed as total flux of BLI (photons/second; radiance photons) ± SE in both in vivo and ex vivo studies.

### Detection of AML cells in circulating blood

To detect leukemic cells in the blood, CD45 leukocyte marker detection by FACS was performed. Red blood cells were lysed from the blood samples according to the instructions of the lysis buffer (eBioscience, TFS). After that, cells were incubated with 10 μL of PE-Cy5 Mouse Anti-Human CD45 (ref. 555484, BD Biosciences) for 20 min in agitation and dark at 4 °C. Cells were washed and re-suspended with PBS-BSA. CD45-positive cells were detected by flow cytometry using FACS Calibur cytometer (BD Biosciences). Data acquisition was analyzed by Cell Quest Pro software (BD Biosciences) and results were expressed as the number of CD45-positive events of a total of 10,000 events ± SE.

### Immunohistochemical staining of leukemic cells in AML affected organs

Immunohistochemical analysis was performed using paraffin-embedded tissue samples as described before [26] using anti-human CD45 antibody (ref. IR75161-2, Dako, Agilent, Santa Clara, CA, USA) to detect infiltration of AML cells and anti-human CXCR4 antibody (ref. AB124824, Abcam, Cambridge, UK) to evaluate CXCR4 expression in tissues. AML infiltration by CD45 detection was evaluated with cellSens Dimension 1.9 software (Olympus, Tokyo, Japan) using the Counter and measurement tool. Results were expressed as the percentage of surface stained ± SE. Stain intensity was calculated using Image J software (1.8.0.172) and the Colour Deconvolution Plugin with the H DAB vector to split the brown staining adjusting the threshold to 150. After that, the “Analyze particles” plugin was used to detect all stained areas and the mean gray value was obtained combining all selected black areas and measuring using the ROI Manager. The intensity value of each analyzed slide was calculated subtracting 255 to the mean gray value obtained by the Image J analysis. Six slides for each treatment type and tissue were analyzed and results were expressed as mean ± SE intensity values. Two independent observers evaluated all samples to made consensus decisions.

### Toxicity analyses in tissues and blood

Hematoxylin and eosin staining was performed in the organs infiltrated by leukemia cells (bone marrow, liver, and spleen), and also in non-affected organs to evaluate possible off-target toxicities of the nanoconjugate (lung, brain, pancreas, heart, and kidney). In addition, plasma glutamic oxaloacetic transaminase (GOT) and glutamic pyruvic transaminase (GPT) enzyme activities, and creatinine levels were determined using commercial kits (ASTL ref. 20764949 322; ALTL ref. 20764957 322; CREJ2 ref. 04810716 190, respectively. Roche Diagnostics), adapted for a COBAS 6000 autoanalyzer (Roche Diagnostics), to evaluate toxicity in tissues such as the liver, kidneys, or heart after T22-GFP-H6-Auristatin treatment in mice.

### Statistical analysis

Statistical analyses were performed in the IBM SPSS Statistics (Release 22.0.0.0, New York, NY, USA). Mann-Whitney *U* test was used in both in vitro and in vivo assays, and any differences were considered statistically significant when the *p* value was < 0.05. IC_50_ were calculated with SigmaPlot (Release 12.0.0.182; Systat Software, Inc., San Jose, CA, USA) using non-linear regression test with Hill-3 parameter adjustment.

## Results

### T22-GFP-H6-Auristatin nanoconjugate synthesis and characterization

**Fig. 1:**
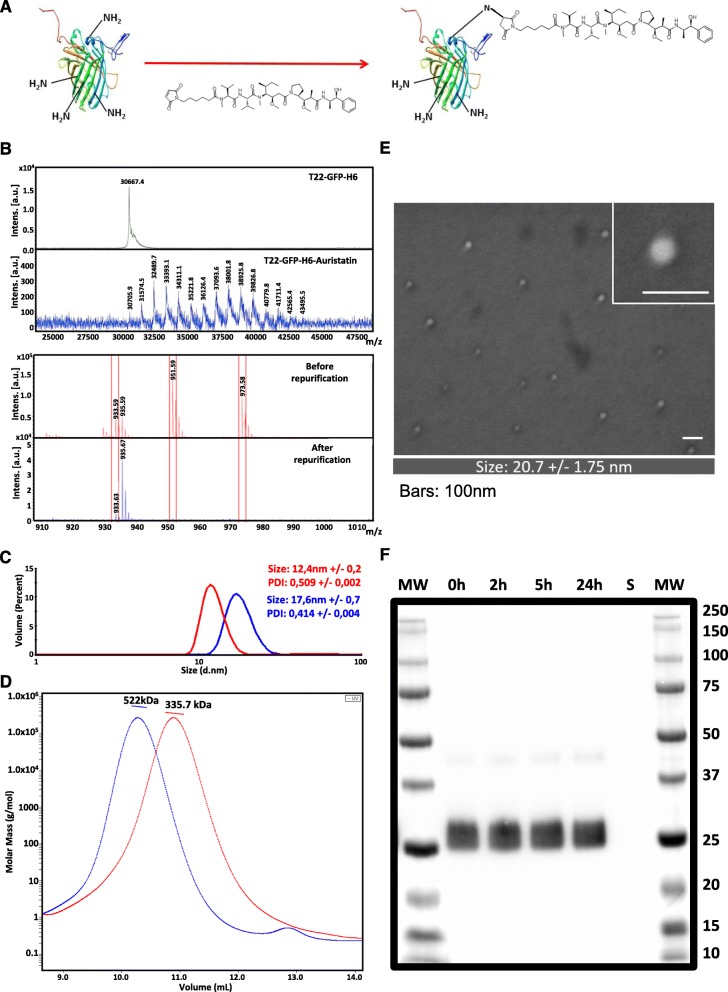
T22-GFP-H6-Auristatin nanoconjugates characterization and stability. **a** T22-GFP-H6 nanoparticle conjugation with maleimide functionalized Monomethyl Auristatin E (MMAE). **b** MALDI-TOF mass spectrometry of wild-type T22-GFP-H6 nanoparticles and T22-GFP-H6-Auristatin nanoconjugates (top) and free MMAE before and after nanoconjugates re-purification (bottom). Each peak corresponds to the covalent addition (+ 911 Da) of a single MMAE molecule (top). Red boxes indicate free Auristatin mass spectrometry spectra (bottom). **c** Hydrodynamic volume size distribution of wild-type T22-GFP-H6 nanoparticles (red) and T22-GFP-H6-Auristatin nanoconjugates (blue) determined by dynamic light scattering. Samples were analyzed in triplicate and data represented as mean +/− SE. PDI indicates polydispersion index. **d** Average molar mass distribution of wild-type T22-GFP-H6 nanoparticles (red) and T22-GFP-H6-Auristatin nanoconjugates (blue) determined by size exclusion chromatography coupled to multi-angle light scattering (SEC-MALS). **e** Representative electron microscopy (FESEM) images of T22-GFP-H6-Auristatin nanoconjugates presented in two magnifications (see inset). Scale bars indicate 100 nm. In the bottom, the quantitative average size of nanoconstructs determined by image analysis and shown as mean ± SE. **f** Proteolytic stability of T22-GFP-H6-Auristatin nanoconjugates in human serum at different incubation times up to 24 h analyzed by western blot immunodetection with a monoclonal anti-His antibody. “S” indicates human serum control. MW, molecular weight; SE, standard error

T22-GFP-H6-Auristatin nanoconjugates (NCs) were generated by covalent reaction of maleimide functionalized MMAE with amine groups of exposed lysines in T22-GFP-H6 nanoparticles (Fig. 1a). Reaction efficacy was then checked by MALDI-TOF mass spectrometry. In this context, MALDI-TOF spectra of T22-GFP-H6-Auristatin NCs showed up to 14 peaks with sequential molecular weight increase of around 911 Da over the wild-type T22-GFP-H6 molecular weight (30.6 kDa) corresponding to the addition of differences number of MC-MMAE molecules (911 Da) (Fig. 1b top). Moreover, low molecular weight spectra showed efficient elimination of non-conjugated free MC-MMAE after nanoconjugates re-purification by IMAC affinity chromatography (Fig. 1b bottom).

Auristatin conjugation resulted in no significant specific fluorescence alteration (data not shown) but in a significant increase of NCs size (17.6 nm) over the wild-type T22-GFP-H6 nanoparticles size (12.4 nm) (Fig. 1c). SEC-MALS analysis indicated an average molar mass increase of 186.3 kDa in T22-GFP-H6-Auristatin NCs (522 kDa) over the wild-type T22-GFP-H6 nanoparticles (335.7 kDa). Considering that each T22-GFP-H6 nanoparticle is generated by the assembling of an average of 11 protein monomers (30.6 kDa each) and the molecular weight of MC-MMAE represents 911 Da, this total molar mass increase over the whole nanoparticle indicates the addition of an average of 18.5 MMAE molecules per protein monomer or 204.5 MMAE molecules per nanoparticle (Fig. 1d). Moreover, we also performed a morphometric characterization of the NC using field emission scanning electron microscopy (FESEM) which allows to visualize the size and shape of our NCs without any staining. The FESEM images (Fig. 1e) showed circular NCs compatible in size with dynamic light scattering data. Finally, the stability of the NC in serum was determined by western blot, showing the absence of degradation for at least 24 h in the blood (Fig. 1f).

### CXCR4-dependent NC internalization and killing of CXCR4 + AML cells *in vitro*

**Fig. 2:**
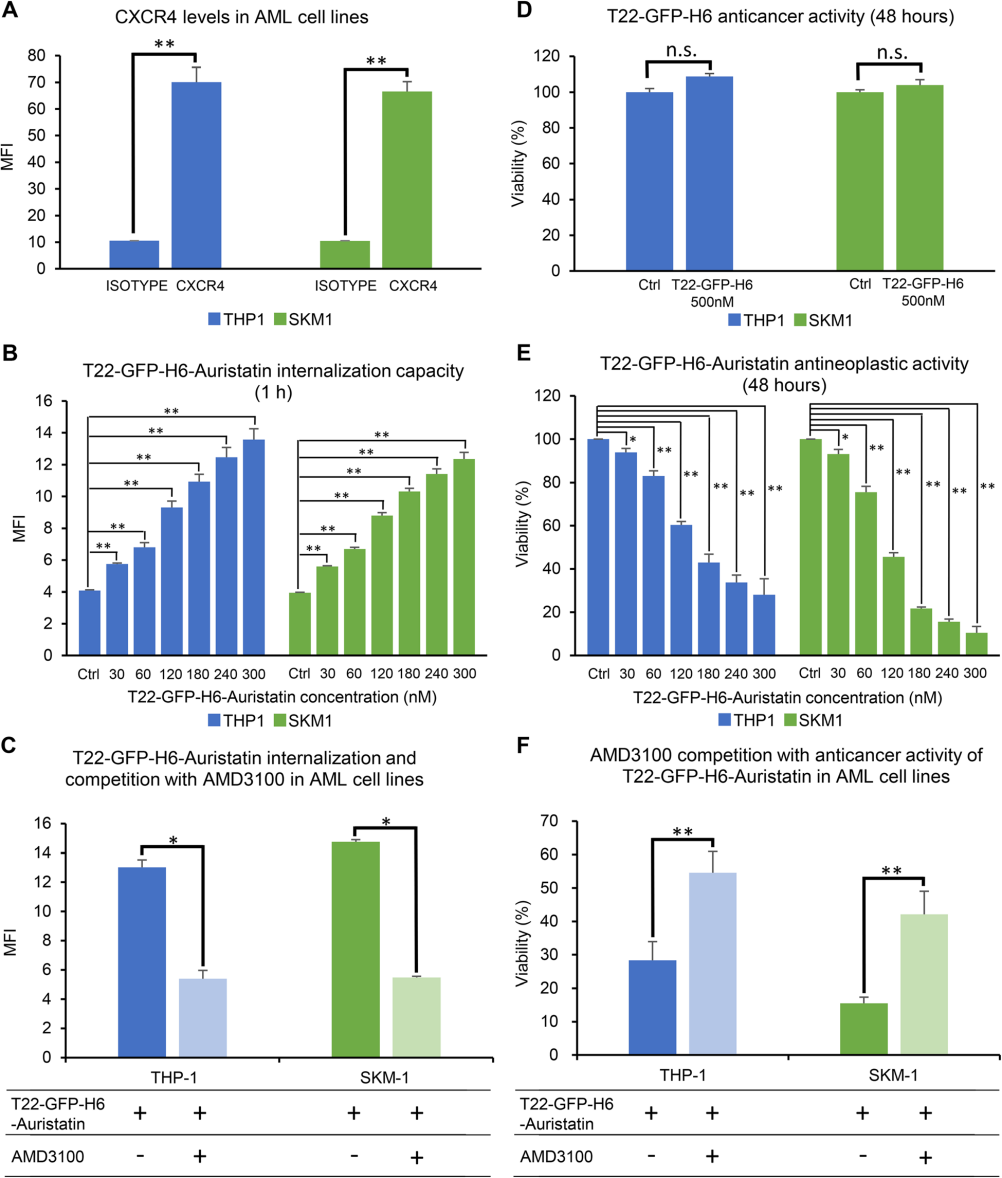
Internalization capacity and antineoplastic activity of T22-GFP-H6 and T22-GFP-H6-Auristatin in AML cell lines. **a** CXCR4 membrane expression of THP-1 and SKM-1 AML cell lines by flow cytometry. **b** Evaluation of T22-GFP-H6-Auristatin internalization, by flow cytometry, in CXCR4+ leukemic cells after 1 h of incubation at different concentrations. **c** Competition assay with AMD3100 (240 nM T22-GFP-H6-Auristatin and 2000 nM AMD3100) to determine the specificity of internalization through CXCR4 receptor. **d** Evaluation of antineoplastic activity of T22-GFP-H6 after 48 h of treatment in AML cell lines performed by XTT assay. **e** Anticancer activity of T22-GFP-H6-Auristatin in AML cell lines after 48 h of incubation by XTT assay. **f** Competition of antineoplastic activity of T22-GFP-H6-Auristatin at 240 nM with AMD3100 at 2000 nM after 48 h exposure by XTT assay. Results are presented as mean ± SE MFI in **a**, **b**, and **c**, and mean ± SE percent of cell viability in **d**, **e,** and **f**. *U* of Mann-Whitney test was used to test differences between groups. In **b** and **e**, T22-GFP-H6-Auristatin treated cells were compared with the buffer treated cells (Ctrl). Statistical significant differences are indicated by * when *p* value < 0.05 and ** when *p* value < 0.001. n.s. means no significance. AML, acute myeloid leukemia; Ctrl, control; MFI, mean fluorescence intensity; SE, standard error

First, CXCR4 levels of THP-1 and SKM-1 cell lines were evaluated by flow cytometry. In this context, high membrane expression of CXCR4 was detected in both tested AML cell lines (THP-1: 70.1 ± 6.7 MFI; SKM-1: 66.6 ± 6.3 MFI) (Fig. 2a). Afterwards, the internalization capacity of the T22-GFP-H6-Auristatin NC was assessed by flow cytometry in these AML cell lines. T22-GFP-H6-Auristatin internalized in a dose-dependent manner after 1 h exposure reaching similar levels in THP-1 and SKM-1 cells, in the 30–300 nM range (Fig. 2b). Finally, a competition assay using AMD3100, a CXCR4 antagonist, showed that the pre-treatment of cells with AMD3100 decreased significantly the internalization capacity of T22-GFP-H6-Auristatin after 1 h exposure (THP-1: 13.0 ± 0.5 vs 5.4 ± 0.2 MFI, *p* = 0.004; SKM-1: 14.8 ± 0.6 vs 5.5 ± 0.1 MFI, *p* = 0.004) (Fig. 2c).

The antitumor activity of T22-GFP-H6-Auristatin was also evaluated *in vitro* in THP-1 and SKM-1 AML cell lines using XTT cell viability assays. First, we analyzed the antineoplastic activity of the unconjugated T22-GFP-H6 nanoparticle at 500 nM. XTT assays showed that T22-GFP-H6 had no anticancer activity in the two tested cell lines (Fig. 2d). In contrast, nanoconjugate had a dose-dependent antineoplastic effect after cell exposure (Fig. 2e), with a IC_50_ of 156.0 and 105.8 nM in THP-1 and SKM-1, respectively. Finally, the competition assays showed that pre-treating the cells with AMD3100 significantly decreased the anticancer activity of T22-GFP-H6-Auristatin in both AML cell lines (THP-1: 54.5 ± 6.3 vs 28.3 ± 5.6% viability, *p* < 0.001; SKM-1: 42.1 ± 6.9 vs 15.5 ± 1.9% viability, *p* < 0.001) (Fig. 2f).

**Fig. 3:**
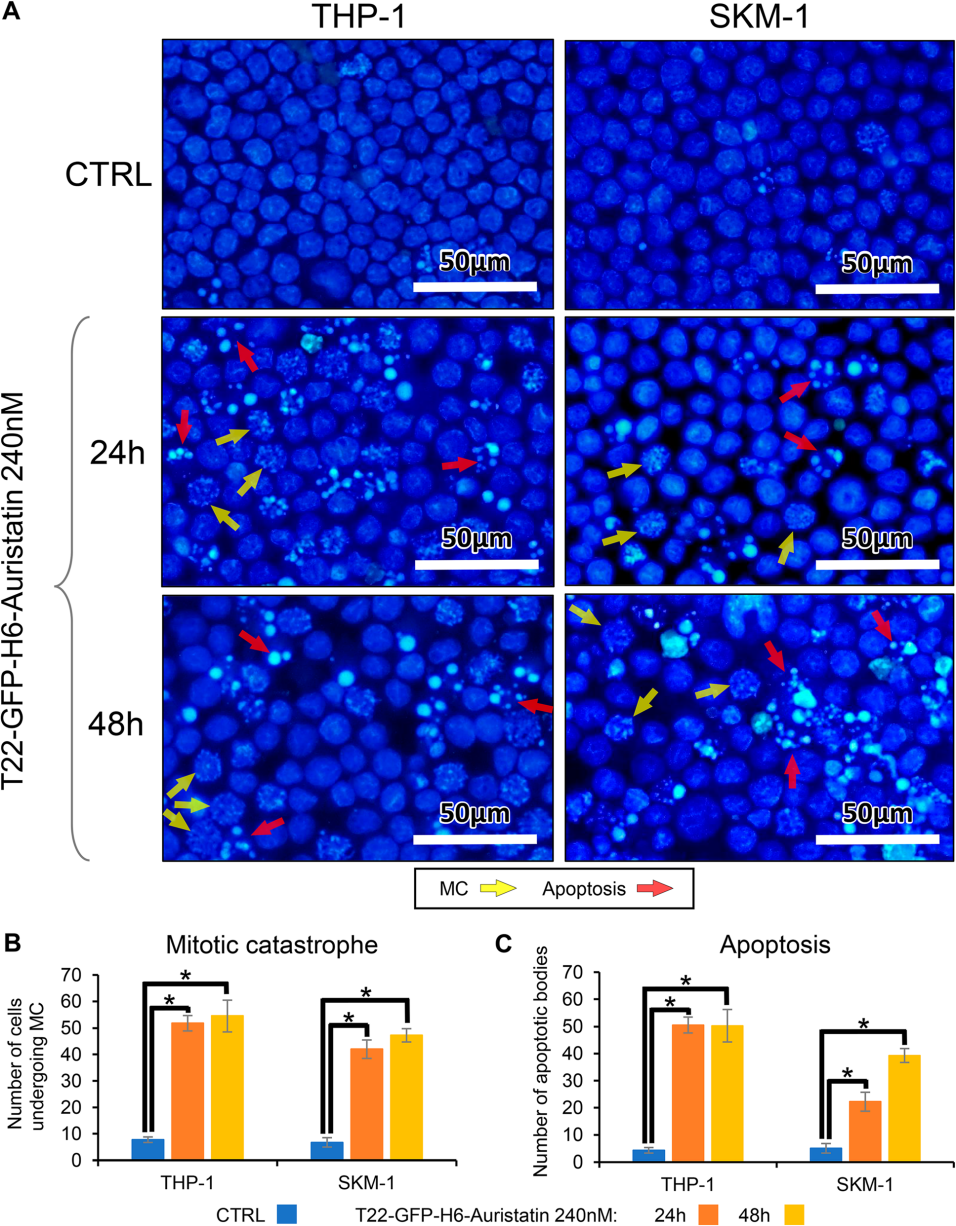
Study of cell death in AML cell lines caused by T22-GFP-H6-Auristatin treatment. Study of the mechanism of T22-GFP-H6-Auristatin-induced cell death. **a** Induction of apoptosis and mitotic catastrophe by T22-GFP-H6-Auristatin at 240 nM for 24 and 48 h of exposure detected by DAPI staining in CXCR4-positive THP-1 and SKM-1 leukemic cell lines. Yellow and red arrows point to MC figures and apoptotic bodies, respectively. **b** Quantitation of the number of cells undergoing mitotic catastrophe triggered by treatment with T22-GFP-H6-Auristatin at 240 nM in THP-1 and SKM-1 for 24 (orange bars) or 48 h (yellow bars). **c** Quantitation of the number of apoptotic bodies in THP-1 and SKM-1 induced by exposure to 240 nM T22-GFP-H6-Auristatin for 24 (orange bars) or 48 h (yellow bars). Results in **b** and **c** are presented as mean ± SE cell number for each cell death event (MC or apoptosis) in all fields analyzed. * indicates significant differences between groups with a *p* value < 0.05. CTRL, control; MC, mitotic catastrophe; SE, standard error

Additionally, the mechanism of cell death induced by T22-GFP-H6-Auristatin was analyzed in both AML cell lines using nuclear staining with DAPI dye (Fig. 3). Cell treatment with 240 nM T22-GFP-H6-Auristatin resulted in the induction of apoptosis and mitotic catastrophe (MC) at 24 and 48 h after cell exposure for both AML cell lines (Fig. 3a). More precisely, MC events were significantly increased after 24 h of incubation with the NC compared to the number of MCs of the CTRL cells in THP-1 (51.8 ± 2.9 vs 7.8 ± 1.0 MCs, *p* = 0.020) and SKM-1 (42.0 ± 3.5 vs 6.8 ± 1.8 MCs, *p* = 0.020) (Fig. 3b). Similarly, the number of apoptosis found in NC-treated cells was increased at 24 h in THP-1 (50.5 ± 5.3 vs 4.3 ± 0.9 apoptotic bodies, *p* = 0.020) and SKM-1 (22.3 ± 4.3 vs 5.0 ± 1.1 apoptotic bodies, *p* = 0.021) compared to the apoptosis observed in buffer-treated cells (CTRL) (Fig. 3c). After 48 h, the number of cell death events were similar to those found at 24 h (Fig. 3b, c). However, in SKM-1, apoptotic bodies increased to reach 39.3 ± 2.2 at 48 h (*p* = 0.020) (Fig. 3c).

### Generation of a CXCR4+ AML model with bone marrow and extramedullar involvement

**Fig. 4:**
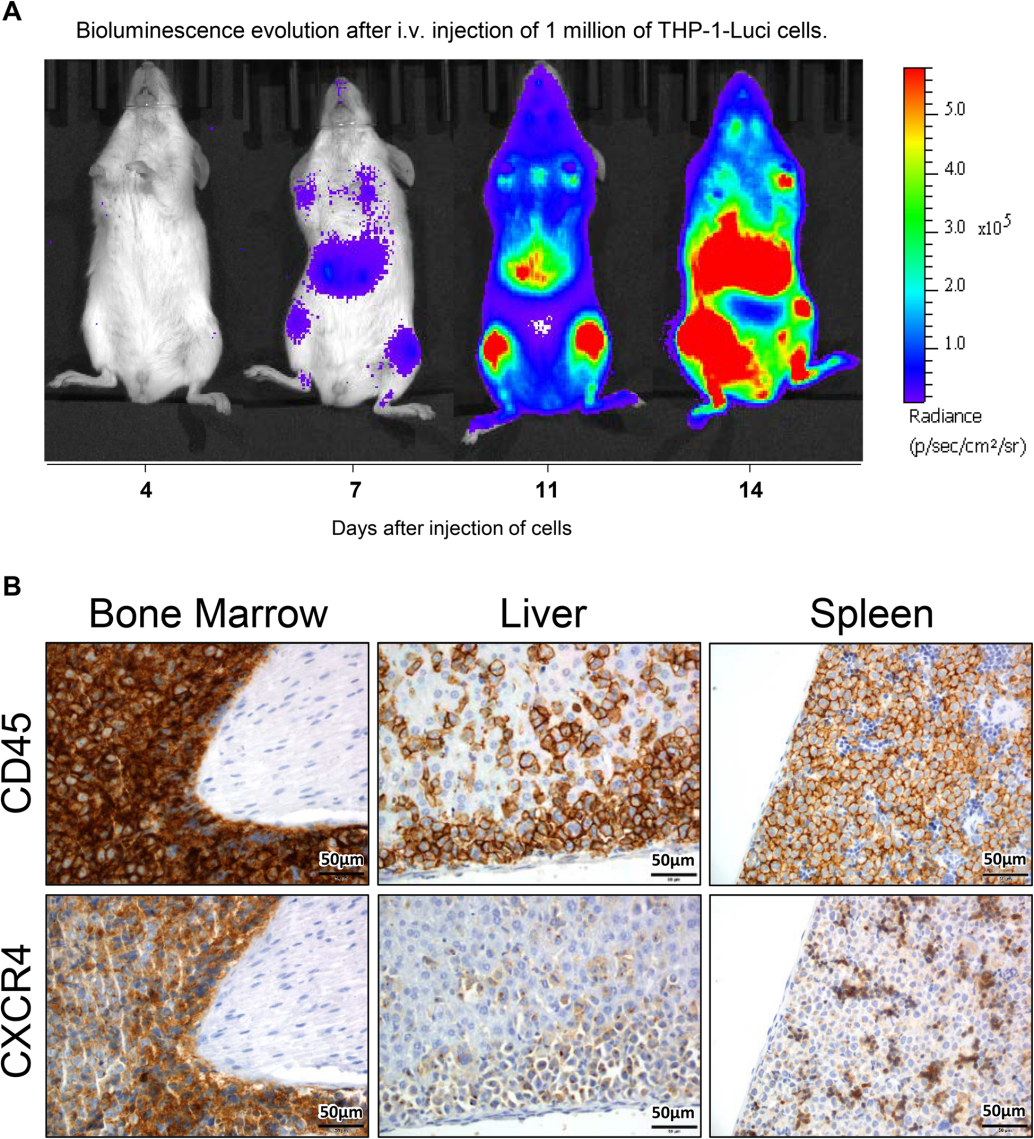
Follow-up of bioluminescence, detection of CD45 and CXCR4 expression in the disseminated AML mouse model. **a** Evolution of bioluminescence along 14 days period after the intravenous (i.v.) injection of CXCR4+ luciferase-transfected THP-1 cells (THP-1-Luci) in NSG mice measuring radiance intensity using the IVIS Spectrum. **b** Detection of CD45 and CXCR4 levels, by IHC, in the bone marrow, liver, and spleen of the leukemic mice 14 days after i.v. injection of THP-1-Luci cells

A disseminated AML mouse model overexpressing CXCR4 and expressing luciferase that allows the follow-up of the fate of the target leukemic cells under therapy was generated. This model was afterwards used to evaluate the anticancer effect of T22-GFP-H6-Auristatin. Model generation consisted in the IV injection of THP-1-Luci cells in immunosuppressed NSG mice. After the injection of 1 × 10^6^ cells, the dissemination of AML can be non-invasively monitored with the IVIS Spectrum equipment (Fig. 4a). In this model, bioluminescence was started to be detected 6 days after injection of cells and until the day of the euthanasia. The increasing burden of cancer growth started giving clear signs of advanced disease or a significant weight loss in mice between days 14 and 15 after tumor cell injection, the time point at which the mice had to be killed to avoid suffering. At necropsy, after mouse euthanasia, tissues were preserved in paraffin and an exhaustive analysis was performed to evaluate CD45 (presence of leukemic cells) and CXCR4 levels by IHC. Expression of both proteins was detected in the bone marrow, liver, and spleen of the mice, therefore generating a disseminated THP-1 AML model displaying high CXCR4 overexpression in leukemia-involved tissues (Fig. 4b). CXCR4 expression was detected not only in the cell surface of the THP-1-Luci cells, but also internalized in endosomes within cells.

### Nanoconjugate reduction of leukemia dissemination in the bone marrow and extramedullar sites

**Fig. 5:**
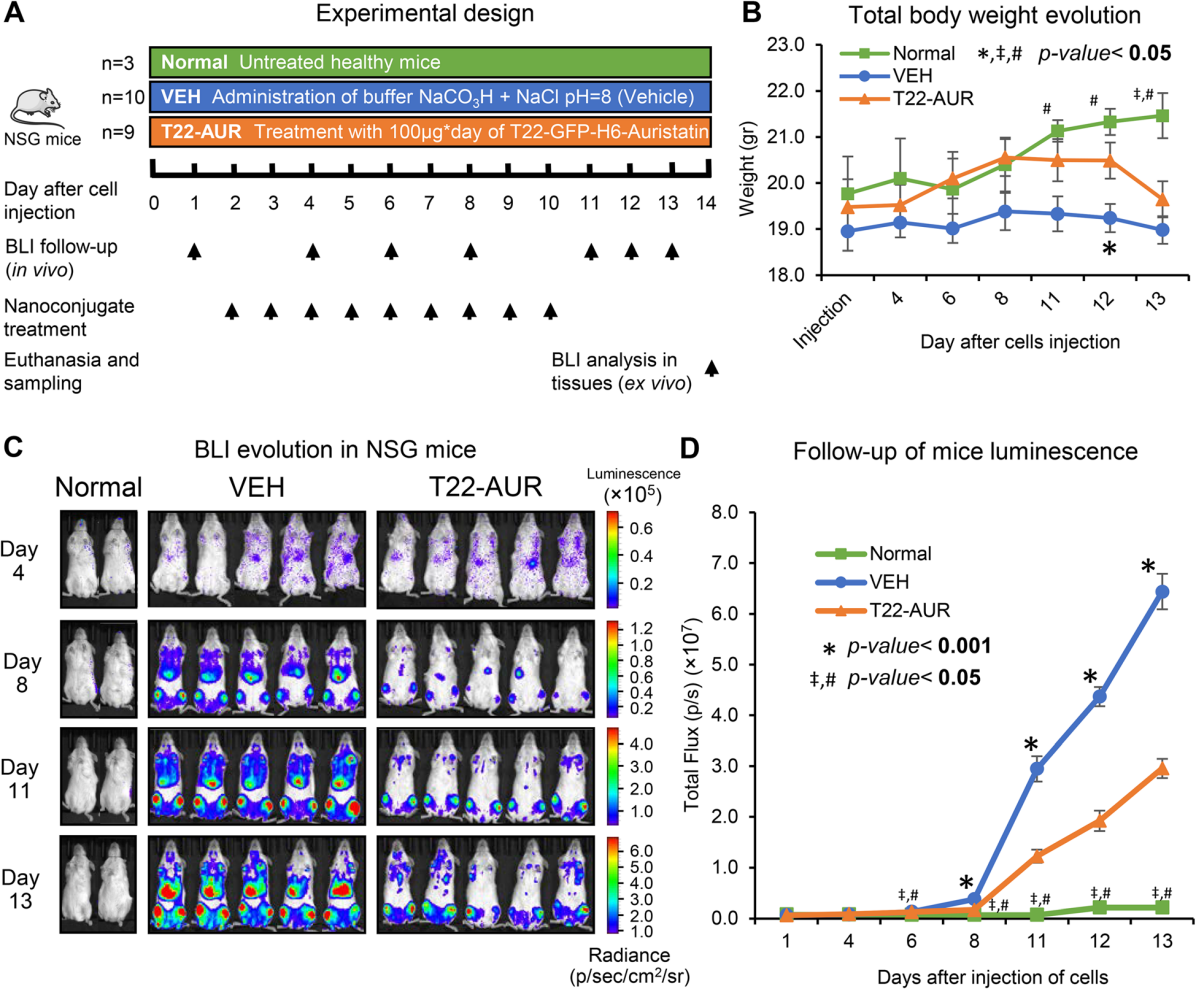
Antineoplastic activity of T22-GFP-H6-Auristatin in a disseminated AML mouse model. **a** Experimental design of the in vivo assay used to assess the antineoplastic activity of T22-GFP-H6-Auristatin. **b** Measurement of total body weight of mice during the experimental time according to the treatment group. Results are presented as mean ± SE body weight in grams. **c** Comparison of evolution of bioluminescence emission in mice treated with vehicle (VEH) or T22-GFP-H6-Auristatin (T22-AUR) and untreated healthy mice (Normal group) the days 4, 8, 11, and 13 after injection of THP-1-Luci cells, measured by the IVIS Spectrum. **d** Follow-up of total body bioluminescence emission of mice treated with T22-GFP-H6-Auristatin (T22-AUR) or vehicle (VEH) and untreated healthy mice (Normal) during all the experiment, analyzed in IVIS Spectrum. Results are presented as mean ± SE luminescence values in photons per second (total flux [p/s]). *U* of Mann-Whitney test was used to assess significant differences between groups in these studies (**b**, **d**), and these differences were considered statistically significant when the *p* value was lower than 0.05. * indicates differences between the VEH and T22-AUR groups, # between the Normal and VEH groups, and ‡ between the Normal and T22-AUR groups. BLI, bioluminescence; T22-AUR, T22-GFP-H6-Auristatin group; VEH, vehicle group.;SE, standard error

The antineoplastic activity of the T22-GFP-H6-Auristatin NC was assessed in vivo, in the disseminated THP-1 AML model described above. The experimental design is depicted in Fig. 5a. On day 12 after AML cell injection, the VEH group presented a significant decrease in body weight in comparison to the T22-AUR-treated group (19.2 ± 0.3 vs 20.5 ± 0.4 gr. resp. *p* = 0.018) (Fig. 5b). In contrast, the mouse weight in the Normal group, not bearing cancer and being untreated, increased significantly as compared to the T22-AUR group (on day 13) or to the VEH group (days 11–13) (Fig. 5b). From day 8 and until the end of the experiment, T22-GFP-H6-Auristatin-treated group showed a total luminescence intensity in the whole animal significantly lower than that observed in the VEH group (Fig. 5c, d). These observed differences between groups progressively increased from the day 8 until 13, even during the 3 days lapse after the last dose of T22-GFP-H6-Auristatin, which was administered on day 10. The background BLI detected in the Normal group was significantly lower than that registered in VEH or T22-AUR treated groups from day 6 after cell injection until the end of the experiment.

In addition, we measured ex vivo the bioluminescence in the bone marrow and extramedullar sites separately (liver and spleen). Luminescence emission by hindlimbs measured leukemic cell load in the bone marrow, whereas luminescence emission by abdominal mouse sections, which included the liver and spleen, measured the leukemic burden at extramedullar sites. A reduction in bioluminescence in T22-GFP-H6-Auristatin-treated, as compared to the VEH-treated group was evident soon after treatment initiation, for full-body emission, bone marrow emission, and extramedullar site emission, all measured sites becoming highly significant at day 8 after treatment initiation, a significance that was maintained until the end of the experiment (day 13) (Table 1).

**Table 1:** Bioluminescence intensity levels emitted in different body sections of mice along the experimental time

		Day after i.v. injection of cells						
	Treatment	1	4	6	8	11	12	13
Full body	VEH	6.61E+05	7.91E+05	1.29E+06	3.76E+06	2.95E+07	4.37E+07	6.44E+07
	T22-AUR	6.33E+05	8.43E+05	1.25E+06	1.65E+06	1.22E+07	1.92E+07	2.96E+07
	*p* value	0.4624	0.5675	0.3904	**0.0002**	**0.0003**	**0.0002**	**0.0002**
Hindlimbs section (bone marrow)	VEH	2.03E+05	2.80E+05	4.94E+05	1.57E+06	1.54E+07	2.21E+07	2.93E+07
	T22-AUR	1.95E+05	3.01E+05	4.67E+05	7.20E+05	8.34E+06	1.25E+07	1.80E+07
	*p* value	0.4375	0.4377	0.1777	**0.0002**	**0.0043**	**0.0009**	**0.0033**
Abdominal section (extramedullar)	VEH	1.94E+05	2.51E+05	4.76E+05	1.51E+06	9.13E+06	1.52E+07	2.70E+07
	T22-AUR	1.77E+05	2.88E+05	4.44E+05	5.39E+05	1.41E+06	2.45E+06	4.70E+06
	*p* value	0.2699	0.1651	0.5401	**0.0002**	**0.0002**	**0.0002**	**0.0002**

Levels of luminescence detected using the IVIS Spectrum in mice treated with vehicle (VEH) or T22-GFP-H6-Auristatin in full body, hindlimbs section (bone marrow), and in abdominal section (including liver and spleen) of mice during the experiment. Results are presented by mean total flux [photons/second] of radiance detected in mice for each group. *U* of Mann-Whitney test was used to assess the significance of the differences between groups. These differences were considered statistically significant when the *p* value was lower than 0.05 (bold values)

BLI bioluminescence, *i.v.* intravenously, *T22-AUR* T22-GFP-H6-Auristatin group, *VEH* vehicle group

### Nanoconjugate reduction of AML cell burden in the bone marrow, circulating blood, liver, and spleen

**Fig. 6:**
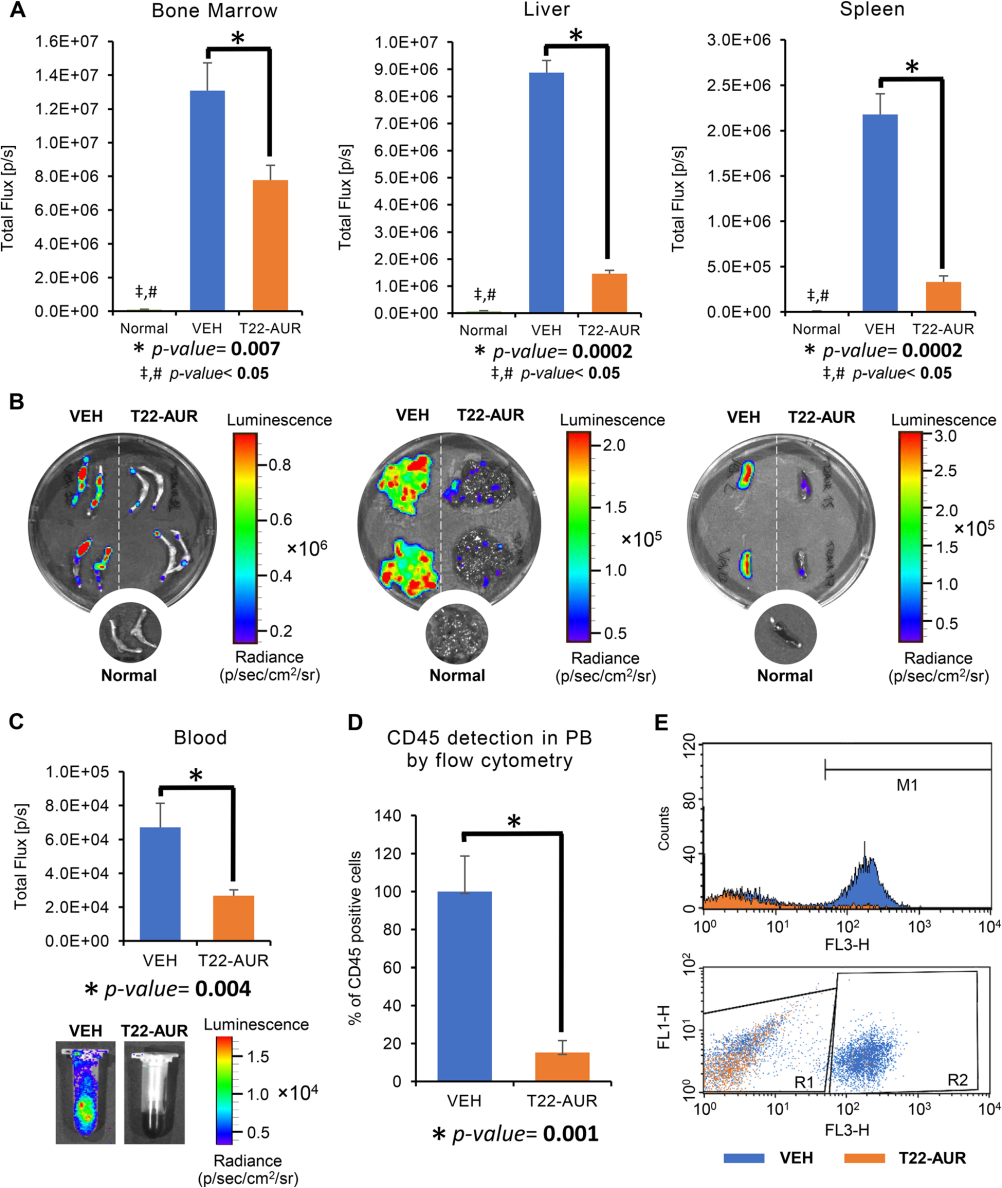
Antineoplastic activity of T22-GFP-H6-Auristatin in the bone marrow, circulating blood, and affected extramedullar sites ex vivo. **a** Comparison of bioluminescence emission between the T22-GFP-H6-Auristatin-treated (T22-AUR, orange bars), vehicle-treated (VEH, blue bars), and untreated healthy mice (Normal, green bars) groups in the bone marrow, liver, and spleen. **b** Bioluminescence Images comparing leukemia dissemination in the bone marrow, liver, and spleen of mice treated with the nanoconjugate (T22-AUR) or vehicle (VEH), and untreated healthy mice (Normal). **c** Comparison of levels of bioluminescence emitted by circulating blood between groups (VEH vs T22-AUR) detected ex vivo at the end of the experiment. **d** Detection of CD45-positive cells in the peripheral blood by flow cytometry in mice treated with vehicle (VEH, blue) or T22-GFP-H6-Auristatin (T22-AUR, orange). Results are presented as percentage, normalizing the number of CD45-positive cells found in a sample by the mean of CD45-positive cells found in all vehicle-treated mice. **e** Histogram and dot plot representations of FL3-H fluorescence detection in a mouse blood sample treated with vehicle (VEH, blue) overlapped with another treated with T22-GFP-H6-Auristatin (T22-AUR, orange). M1 and R2 sections indicate CD45-positive cells, whereas R1 section indicates CD45-negative cells. Results in studies **a** and **c** are presented as mean ± SE luminescence values in photons per second (total flux [p/s]), and as mean ± SE of the percent of CD45-positive cells in **d**. *U* of Mann-Whitney test was used in studies **a**, **c**, and **d** to assess significant differences and are presented in the figures with an * to compare between the VEH and T22-AUR groups, # between the Normal and VEH groups, and ‡ between the Normal and T22-AUR groups. The differences between groups were considered significant when the *p* value was lower than 0.05. PB, peripheral blood; T22-AUR, T22-GFP-H6-Auristatin group; VEH, vehicle group; SE, standard error

The anticancer effect of the NC was assessed ex vivo, measuring luminescence emission by leukemic cells in all AML affected organs as well as in circulating blood at the end of the experiment. Based on these data, T22-GFP-H6-Auristatin treatment significantly decreased the dissemination of leukemic cells to the bone marrow (7.8E+06 ± 8.7E+05 vs 1.3E+07 ± 1.4E+06(p/s), *p* = 0.007), liver (1.5E+06 ± 1.2E+05 vs 8.9E+06 ± 4.5E+05(p/s), *p* = 0.0002), and spleen (3.3E+05 ± 6.8E+04 vs 2.2E+06 ± 2.3E+05(p/s), *p* = 0.0002) as compared to the VEH group (Fig. 6a, b). BLI levels emitted by the Normal group were significantly lower than the other groups (VEH and T22-AUR) in the bone marrow, liver, and spleen (Fig. 6a, b). In addition, blood samples were analyzed with two different techniques to evaluate the anticancer activity of the nanoconjugate on circulating leukemic cells. In the T22-AUR group the luminescence emitted by AML cells circulating in the blood was lower than in the VEH group (2.7E+04 ± 3.5E+03 vs 6.7E+04 ± 1.4E+04(p/s), resp. *p* = 0.004) (Fig. 6c). In addition, CD45-positive cells (a surface marker of AML cells and therefore of THP-1 cells) were also detected by flow cytometry in the blood. Consistently with the observed luminescence results, the relative percentage of CD45-positive cells was significantly lower in the T22-AUR group than in the VEH group (18.7 ± 6.4 vs 100 ± 15.2 %, *p* = 0.001) (Fig. 6d, e). Therefore, in the CXCR4+ disseminated THP-1 model, T22-GFP-H6-Auristatin treatment reduces the leukemic cell burden in the bone marrow and circulating blood, blocking also the leukemic spread to the liver and spleen.

### NC anticancer effect measured as organ affectation by CD45 cells and associated toxicity

**Fig. 7:**
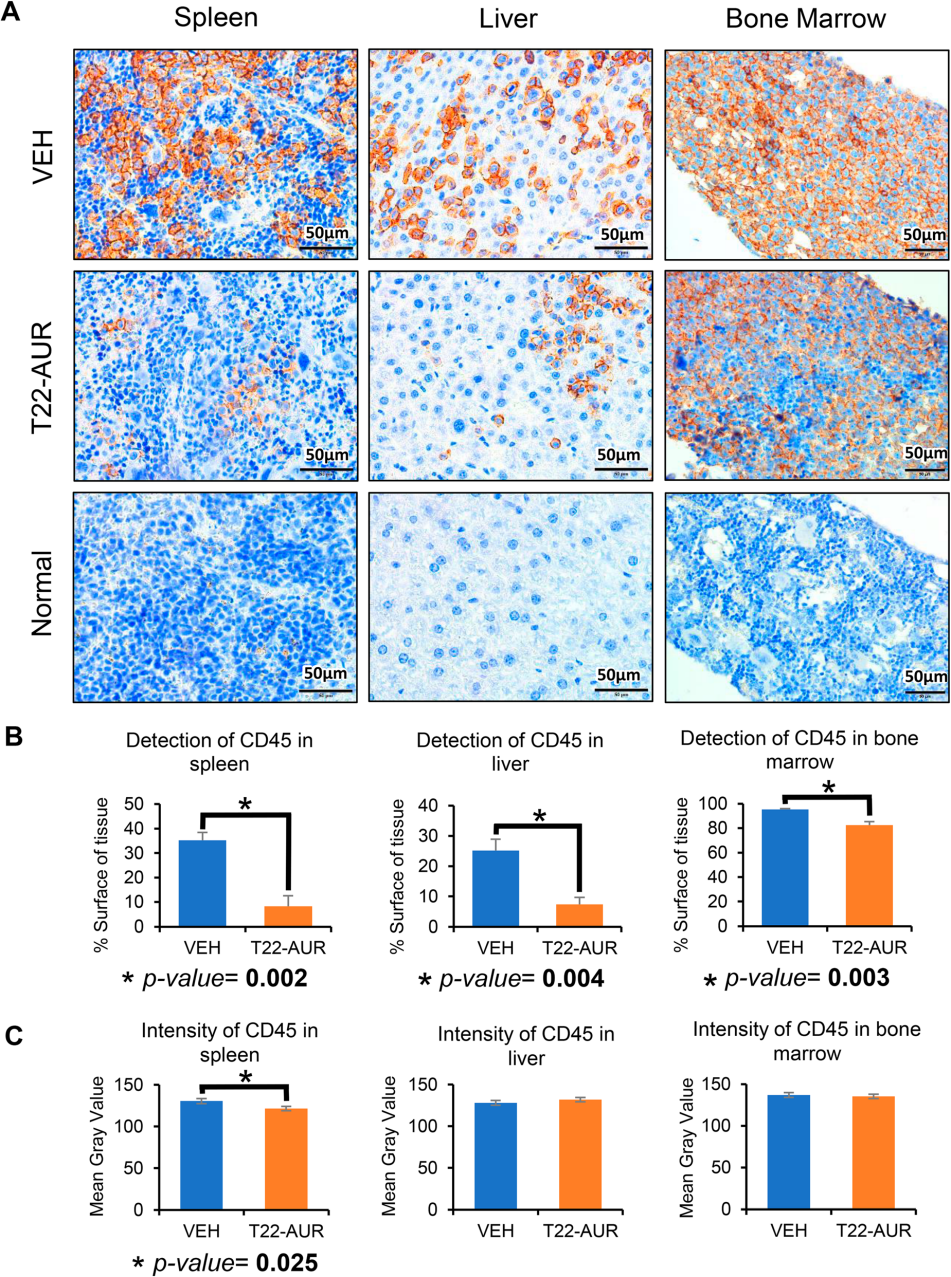
Detection of CD45-positive cells in the leukemia-infiltrated tissues after treatment with T22-GFP-H6-Auristatin. **a** Detection of CD45-positive cells by IHC in the bone marrow and extramedullar organs (spleen and liver) of mice treated with vehicle (VEH) or T22-GFP-H6-Auristatin (T22-AUR) 14 days after injection of CXCR4+ THP-1-Luci cells, and comparison with CD45-positive cell detection in same tissues of a normal non-leukemic mouse. **b** Quantification of the CD45 detection by IHC in the spleen, liver, and bone marrow in mice treated with vehicle (VEH, blue bars) and T22-GFP-H6-Auristatin (T22-AUR, orange bars). This quantification is presented as mean ± SE percentage of stained surface with CD45 antibody in the tissue of all mice. **c** Quantitation of CD45 staining intensity by IHC in the liver, spleen, and bone marrow in mice treated with vehicle (VEH, blue bars) or T22-GFP-H6-Auristatin (T22-AUR, orange bars). Results are presented as the mean ± SE of the mean gray value obtained in Image J (see the “Methods” section). Significant differences were indicated by * when the *p* value was lower than 0.05, using *U* of Mann-Whitney test between groups. T22-AUR, T22-GFP-H6-Auristatin group; VEH, vehicle group; SE, standard error

**Fig. 8:**
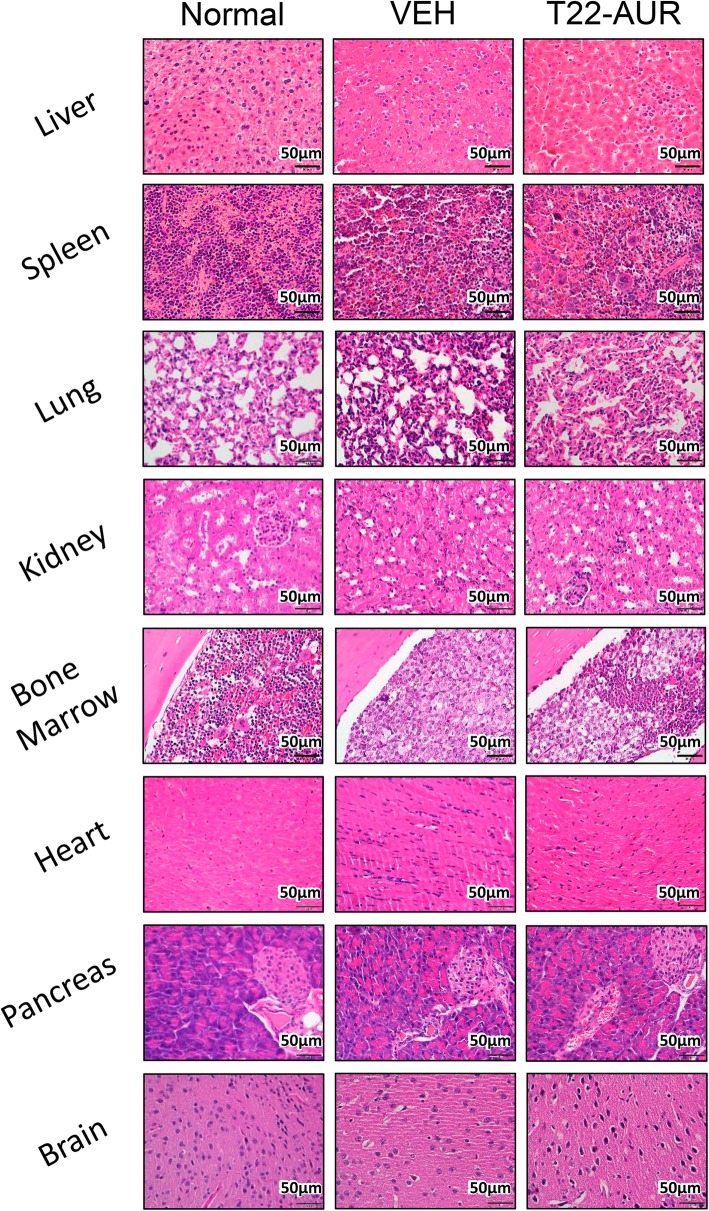
Hematoxylin and eosin staining in leukemia-infiltrated tissues after treatment with T22-GFP-H6-Auristatin. Hematoxylin and eosin staining of the liver, spleen, bone marrow, lung, pancreas, brain, heart, and kidneys of mice treated with vehicle (VEH) or T22-GFP-H6-Auristatin (T22-AUR) 14 days after the injection of CXCR4+ THP-1-Luci cells in NSG mice, compared with the stained tissues of a healthy mouse (Normal). T22-AUR, T22-GFP-H6-Auristatin group; VEH, vehicle group

We analyzed the capacity of the NC to block AML involvement in the spleen, liver, and bone marrow (hindlimbs) using IHC anti-CD45 staining in paraffin-embedded tissues to detect THP-1 AML cells. The percent of positive CD45 within tissue sections was significantly lower in the T22-AUR group than the VEH group in the spleen (8.3 ± 4.2 vs 35.2 ± 8.3%, resp. *p* = 0.002), liver (7.3 ± 2.4 vs 25.2 ± 3.7%, resp. *p* = 0.004), and bone marrow (82.6 ± 2.8 vs 95.3 ± 0.9%, resp. *p* = 0.003) (Fig. 7a, b). In addition, CD45 staining intensity exhibits a similar level between groups in the liver and bone marrow, whereas the spleen shows a significant reduction in the T22-AUR group compared to the VEH-treated group (122 ± 1.8 vs 131 ± 2.7 *p* = 0.025, respectively) (Fig. 7c). Finally, we performed hematoxylin and eosin staining of the three AML affected organs such as the spleen, liver, and bone marrow, and the off-target organs, including the lung, kidney, heart, pancreas, and brain. We did not observe any histological alteration in normal cells found in these organs, indicating a lack of toxicity in these tissues (Fig. 8). In addition, GOT, GPT, and creatinine levels were determined in plasma to evaluate the toxicity, mainly in the liver, kidneys and heart. No significant differences were found in these biochemical parameters between mice treated with 9 doses of T22-GFP-H6-Auristatin compared to buffer treated mice (VEH) (Additional Fig. 1). Thus, T22-GFP-H6-Auristatin treatment decreased the amount of CD45 AML cells detected in the spleen, liver, and bone marrow, validating the reduction of leukemic cell burden in these organs, without inducing toxicity on normal organs.

## Discussion

We developed a novel targeted drug delivery strategy for acute myeloid leukemia (AML) treatment. Repeated administration of the T22-GFP-H6-Auristatin nanoconjugate (NC) in a CXCR4+ THP-1 AML mouse model achieved potent anticancer activity, by significantly reducing the leukemic cell burden in the bone marrow and circulating blood, and by inducing a potent blockade of leukemic cell spread to extramedullar organs. These results were obtained by measuring the bioluminescence emitted by the CXCR4+ luciferase+ AML cells at each affected site, findings that were validated observing a reduction of CD45+ AML cells in the liver and spleen at the end of treatment. This potent blockade of leukemic dissemination was achieved using a NC dosage that did not induce any histological alteration or toxicity on normal organs; therefore, this nanoconjugate displays a high therapeutic index.

Our results show the importance of using a disseminated model for preclinical AML drug development, displaying bone marrow homing, circulating leukemic cells in the blood, and AML cell involvement at extramedullar sites. The luminescence emitted by luciferase+ AML cells allows the non-invasive follow-up of the response of target CXCR4+ leukemic cells to NC treatment in each clinically relevant site. This model outperforms most current models used to evaluate the effectiveness of novel drugs or therapeutic approaches in AML therapy. Thus, many authors evaluate only antitumor activity measuring subcutaneous (SC) tumor size reduction in mouse models [27–30], which cannot evaluate the relevant drug effect on AML cells anchored in the bone marrow [6]. Other groups complement the SC model assessment with orthotopic or patient-derived PDX models [31–36]; however, the lack of luciferase expression in most models prevents the direct measurement of the drug effect on AML cells affecting the bone marrow, liver, or spleen [10].

We explain the potent antineoplastic effect obtained in the bone marrow, circulating cells, and extramedullar sites without associated toxicity, in our model, by the achievement of a high selectivity by the T22-GFP-H6-Auristatin NC in CXCR4+ AML cell killing, and the consequent elimination of leukemic stem cells (LSCs). Thus, CXCR4+ AML cells can be considered LSCs based on their association with bone marrow homing, circulation in the blood, and extramedullar dissemination, all associated with poor prognosis [2, 5, 20]. Two additional reasons also support the notion that this NC achieves selective killing of LSCs, without having an impact on normal cells:

1. Selectivity in CXCR4+ AML cell killing exploits the high overexpression of CXCR4 in LSCs as compared to normal HSCs, as previously described [6, 37], leading to the specific internalization and selective delivery of the conjugated drug to CXCR4+ AML cells in all clinically relevant sites, since our THP-1 model highly express the CXCR4 receptor in the AML cell membrane in BM and extramedullar sites. This selective effect is also supported by the in vitro THP-1 and SKM-1 AML cells results, in which the NC showed a strict selectivity and CXCR4 dependence in internalization and cell killing of CXCR4+ AML cells.
2. Ability of the NC payload, Auristatin E, for killing quiescent cancer cells, as a microtubule destabilizing agent, through the induction of mitotic catastrophe and apoptosis as described [23, 24], which particularly, in our model, eliminates the LSCs that enter quiescence after homing in the BM [6, 20]. This effect may overcome the limited effectiveness of current chemotherapy that blocks AML cells in division but induces life-threatening adverse effects [38], being unable to kill quiescent LSCs. This is likely the reason why Auristatin E is extensively used in antibody-drug conjugates under development, one of which, Brentuximab vedotin, is currently used in the clinic to treat CD30+ Hodgkin’s lymphoma and anaplastic large-cell lymphoma patients [39].

We also think these arguments, and particularly their cancer-selective biodistribution, underlie our expectation that this nanoconjugate may outperform the efficacy of drugs used in the clinic or being under development as we following explain. Thus, the NC selectivity in CXCR4+ AML cell uptake, and selective elimination by the payload drug, differs from the molecularly targeted small drugs that inhibit proteins involved in maintaining the cancer stem cell phenotype (e.g. Wnt, Hedgehog NFkB). The passive diffusion of small drugs to all cells implies a lack of selectivity in their biodistribution to cancer sites; therefore, the achievement of therapeutic concentrations in cancer cells is limited by associated toxicity in normal tissues [40, 41].

The direct killing of CXCR4+ quiescent AML blasts in the BM reached by our nanoconjugate differs also from the effect of low molecular weight inhibitors of the CXCR4 receptor, which in AML are used in combination with chemotherapy. CXCR4 inhibitors mobilize AML blasts from BM to the bloodstream, losing their quiescence, and becoming cycling cells that chemotherapy targets. Nevertheless, AML cell mobilization induces hyper-leucocytosis that could, unexpectedly, generate extramedullar dissemination [5].

Regarding strategies similar to our targeted drug delivery approach, no antibody-drug conjugates (ADCs) targeting CXCR4 have been introduced in the clinic so far. We believe that our protein-based nanoparticle could, however, improve the reported biodistribution described for ADCs, reaching only 0.01–0.1% of the injected dose in cancer cells [42]. In contrast, we have reported that T22-GFP-H6 nanoparticles and the derivate NCs achieve around 85% of the administered dose in tumor tissues, both in solid tumors [22, 43] and hematological neoplasias [26].

Finally, because of the reported overexpression of CXCR4 in AML cells which associates with chemotherapy resistance, LSC development, relapse, and minimal residual disease in AML patients [1–4, 11, 44–47], we believe this nanoconjugate could be used to treat resistant AML. Thus, in the near future, we plan to develop disseminated AML models resistant to therapy to evaluate this hypothesis.

## Conclusions

In summary, we developed a novel nanoconjugate for targeted drug delivery of Auristatin E that achieves a significant reduction of the leukemic cell burden in the bone marrow and circulating blood, and a potent blockade of leukemic cell spread to extramedullar organs in a CXCR4+ AML model. These results prove the high selectivity of the nanoconjugate in controlling AML dissemination without associated toxicity and, importantly, that CXCR4+ AML cells are a relevant target for clinical therapy.

## Supplementary information

**Additional file 1: Figure S1**. GOT, GPT and creatinine levels in plasma after treatment with T22-GFP-H6-Auristatin. Determination of GOT, GPT (**A**) and Creatinine (**B**) in plasma of mice treated with 9 doses of vehicle (VEH) or T22-GFP-H6-Auristatin (100μg/dose) (T22-AUR) 14 days after the injection of THP-1-Luci cells in NSG mice. Results are presented as mean ± SE enzyme activity in U/L for GOT and GPT (**A**) and mean ± SE creatinine levels in mmol/L (**B**). GOT, oxaloacetic transaminase; GPT, glutamic pyruvic transaminase; T22-AUR, T22-GFP-H6-Auristatin group; VEH, Vehicle group. SE, standard error.

## Data Availability

All data generated or analyzed during the current study are available from the corresponding author on reasonable request.

## Abbreviations

ADC: Antibody-drug conjugate
AML: Acute myeloid leukemia
BLI: Bioluminescence
BM: Bone marrow
BSA: Bovine serum albumin
CRC: Colorectal cancer
CXCL12: C-X-C motif chemokine 12
CXCR4: C-X-C chemokine receptor type 4
FACS: Fluorescence-activated cell sorting
GFP: Green fluorescence protein
GOT: Oxaloacetic transaminase
GPT: Glutamic pyruvic transaminase
HSC: Hematopoietic stem cell
IHC: Immunohistochemistry
IV: Intravenously
LSC: Leukemic stem cell
MALDI-TOF: Matrix-Assisted Laser Desorption/Ionization - Time-Of-Flight
MC: Mitotic catastrophe
MC-MMAE: Maleimide conjugated monomethyl Auristatin E
MFI: Mean fluorescence intensity
MMAE: Monomethyl Auristatin E
NC: Nanoconjugate
NSG: NOD-scid IL2Rgammanull
PBS: Phosphate-buffered saline
SC: Subcutaneous
SE: Standard error
SEC-MALS: Size exclusion chromatography – multi-angle light scattering
