# Supplementary figures and images for: An Auristatin nanoconjugate targeting CXCR4+ leukemic cells blocks acute myeloid leukemia dissemination
Source: J Hematol Oncol. 2020 Apr 15;13:36. doi: 10.1186/s13045-020-00863-9 (PMC7160905; doi:10.1186/s13045-020-00863-9)

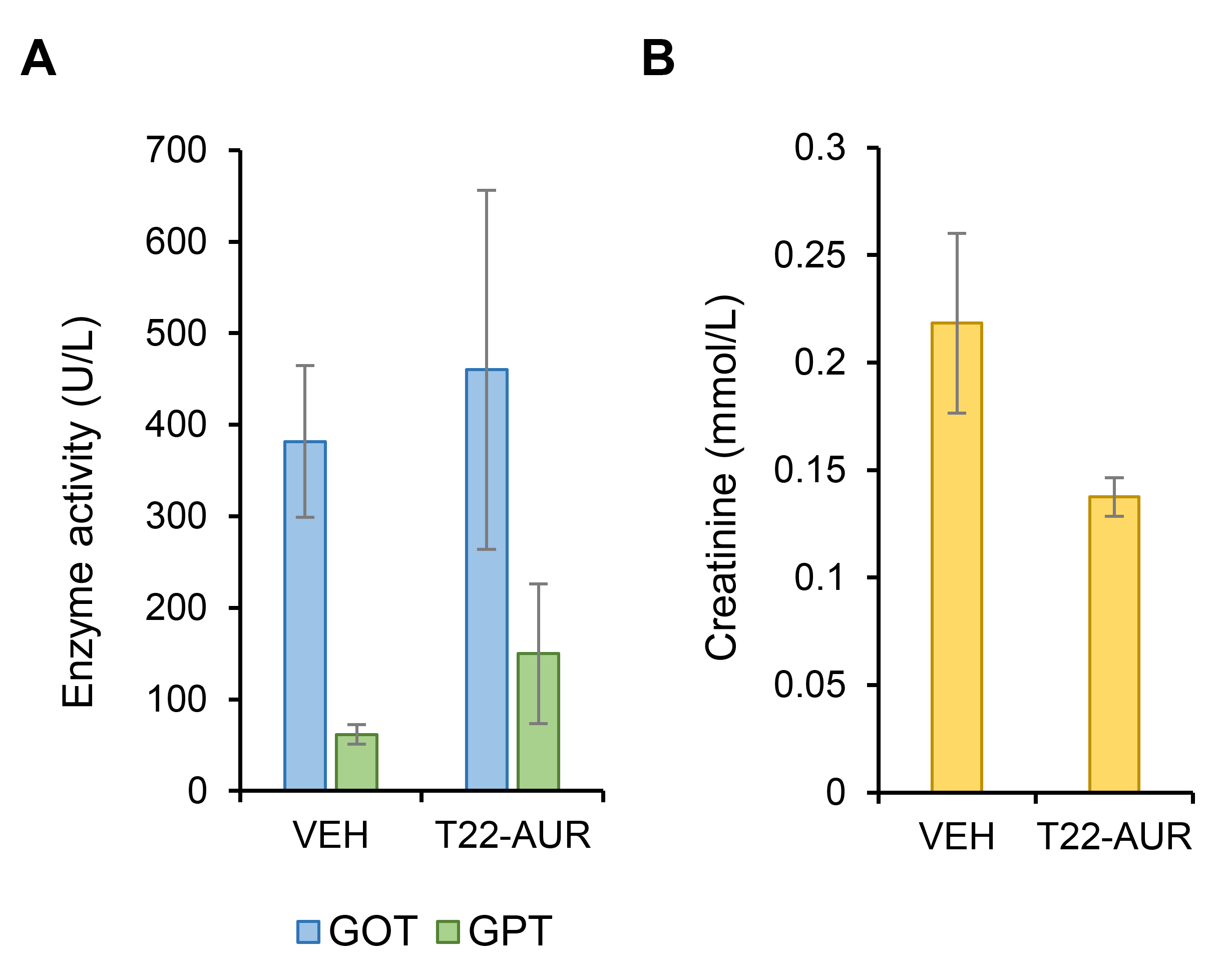

Supplement: Supplementary file 1 — Additional file 1: Figure S1. GOT, GPT and creatinine levels in plasma after treatment with T22-GFP-H6-Auristatin. Determination of GOT, GPT (A) and Creatinine (B) in plasma of mice treated with 9 doses of vehicle (VEH) or T22-GFP-H6-Auristatin (100μg/dose) (T22-AUR) 14 days after the injection of THP-1-Luci cells in NSG mice. Results are presented as mean ± SE enzyme activity in U/L for GOT and GPT (A) and mean ± SE creatinine levels in mmol/L (B). GOT, oxaloacetic transaminase; GPT, glutamic pyruvic transaminase; T22-AUR, T22-GFP-H6-Auristatin group; VEH, Vehicle group. SE, standard error. [file 13045_2020_863_MOESM1_ESM.tif]
